# The Novel Histological Prostatic Inflammation Score Helps Defining the Association Between Stromal and Glandular Inflammation with the Risk of Prostate Cancer at Prostate Biopsy

**DOI:** 10.3390/diagnostics15020166

**Published:** 2025-01-13

**Authors:** Ugo Giovanni Falagario, Francesca Sanguedolce, Angelo Cormio, Antonella Ninivaggi, Marco Finati, Francesco Guzzi, Gian Maria Busetto, Carlo Bettocchi, Daniele Castellani, Giuseppe Carrieri, Luigi Cormio

**Affiliations:** 1 Department of Urology and Renal Transplantation, Policlinico Foggia, University of Foggia, 71122 Foggia, Italy; ugo.falagario@unifg.it (U.G.F.); a.ninivaggi9@gmail.com (A.N.); marco.finati@unifg.it (M.F.); f.guzzi95@gmail.com (F.G.); gianmaria.busetto@unifg.it (G.M.B.); carlo.bettocchi@unifg.it (C.B.); giuseppe.carrieri@unifg.it (G.C.); luigi.cormio@unifg.it (L.C.); 2 Department of Molecular Medicine and Surgery (Solna), Karolinska Institutet, 171 76 Stockholm, Sweden; 3 Pathology Unit, Policlinico Foggia, University of Foggia, 71122 Foggia, Italy; francesca.sanguedolce@unifg.it; 4 Department of Urology, Azienda Ospedaliero-Universitaria Ospedali Riuniti Di Ancona, Università Politecnica delle Marche, Via Conca 71, 60126 Ancona, Italy; castellanidaniele@gmail.com; 5 Department of Urology, Bonomo Teaching Hospital, 76123 Andria, Italy

**Keywords:** prostate cancer, prostatic inflammation, prostate biopsy, lower urinary tract symptoms, benign prostatic obstruction

## Abstract

**Background:** There is emerging evidence of an inverse association between prostatic inflammation (PI) and prostate cancer (PCa) diagnosis and outcome. The Irani score, a validated system that scores PI according to the grade of stromal infiltration (Irani G) and the aggressiveness of glandular infiltration (Irani A), has indeed been found to be inversely associated with PCa diagnosis and outcome, but the presence of two categories (G and A) makes the performance of this score suboptimal. This study aimed to determine whether a novel prostatic inflammation score (PIS) that combines Irani G and A scores better defined the risk of being diagnosed with PCa at prostate biopsy (PBx). **Methods:** Between January 2013 and December 2023, the Irani scores were routinely assessed on hematoxylin and eosin-stained PBx cores. The novel PIS was obtained by combining Irani G and A scores by their kernel distribution. PIS 1 included patients who scored G 0–1/A 0–1, PIS 2 those who scored G 2–3/A 0–1, and PIS 3 included those who scored G 0–3/A 2–3. Logistic regression analysis was used to test the association between the novel PIS and the risk of being diagnosed with PCa and clinically significant (cs) PCa at PBx. **Results:** Among the 4620 eligible patients, PCa and csPCa detection rate was 47% and 25%, respectively. Overall, 3088 (66.8%) had low Irani G and 4041 (87.5%) had low Irani A scores. Using PIS, 2971 (64%) were classified as PIS 1, 1070 (23%) as PIS 2, and 579 (13%) as PIS 3. Notably, almost one-quarter of patients had heterogeneous Irani features. Multivariable analysis pointed out a significant association between PIS and the risk of being diagnosed with PCa and csPCa; the higher the PIS, the lower the likelihood of such diagnoses. Limitations included the absence of external validation. **Conclusions:** The novel PIS, easily obtained during routine pathology examination, was significantly associated with the risk of being diagnosed with PCa and csPCa at PBx. While PI seems to be overall protective over PCa, the different types (stromal vs. glandular) of inflammation depicted by PIS seem to express a different risk.

## 1. Introduction

The contribution of inflammation in the development and progression of cancer is well documented, as the oxidative stress induced by inflammatory cells from their response to external stimuli can lead to molecular damage [1], whose continuous alteration characterizes the genesis of cancer. Indeed, tumor-associated inflammation has been shown to promote tumor invasion, migration and metastasis in various cancers [2,3]. It is also well known that infections such as human papillomavirus (HPV) have been associated with the development of urological cancers [4]. Nonetheless, accumulating evidence indicates that inflammation could be protective as the immune system can identify and kill transformed tumor cells that express modified antigens [5] in a phenomenon known as immune surveillance. The current EAU guidelines on prostate cancer (PCa) recommend documentation of the presence, absence and type of inflammation (acute or granulomatous) in prostate biopsy (PBx) cores, as inflammation is commonly found in PBx samples in clinical practice [6]. Several techniques for grading inflammation in PBx cores through immunohistochemistry or gene expression analysis have been described, but they are expensive and not readily available [7,8]. The Irani score, conversely, is carried out on hematoxylin and eosin stains and classifies prostatic inflammation (PI) based on the extension and severity of inflammation cells in the prostate tissue [9]. Specifically, the Irani Grade (G) depicts the extent of stromal infiltration by the inflammatory cells, whereas the Irani Aggressiveness (A) depicts the extent of glandular infiltration. Previous studies have demonstrated an association between postponed low Irani G with and Irani A scores and an increased risk of PCa diagnosis on PBx [10]. Additionally, low Irani G scores have been shown to predict adverse pathology on RP in low-grade PCa [11]. On the other hand, elevated Irani G scores have been linked to an increased incidence of benign prostatic obstruction (BPO) [10,12,13]. Indeed, a recent randomized controlled trial pointed out that the presence of high Irani G scores in PBx specimens may represent a valuable treatment target in patients diagnosed with BPO, given the possibility to significantly reduce it by a six-month course of hexanic extract of Serenoa Repens [14]. Elevated Irani A scores have been linked to higher PSA levels attributed to the rupture of the prostate epithelium [9], but there was no association with BPO [10]. While available evidence suggests a correlation between the Irani scores and prostatic diseases, the presence of two different scores makes it difficult to interpret the results, particularly when they are heterogeneous. In this study, we combined Irani G and A scores to obtain a single and more comprehensive prostatic inflammation score (PIS) and tested its association with PCa diagnosis at PBx.

## 2. Materials and Methods

The data of all consecutive patients undergoing either initial or repeat PBx at a single center between January 2013 and December 2023 due to elevated serum PSA levels and/or a positive or suspicious digital rectal examination (DRE) were recorded in a prospectively maintained dedicated database. Men who had a history of 5-alpha reductase inhibitor (5-ARI) use, previous surgical intervention for BPH, indwelling urethral catheters or PSA levels exceeding 20 ng/mL were excluded from this study. PSA levels were measured in all patients prior to performing DRE and transrectal ultrasound (TRUS); before TRUS, they also underwent uroflowmetry (UFM), gathering maximum flow rate (Qmax) and a post-void residual volume (PVR) assessed by suprapubic ultrasound. Additionally, all patients completed the International Prostate Symptom Score (IPSS) questionnaire to evaluate the presence and severity of lower urinary tract symptoms (LUTS). Using local non-infiltrative anesthesia [15,16], PBx was carried out based on an 18-core standard biopsy template [17] under TRUS guidance (BK Medical Flex Focus 500, BK Medical ApS, Herlev, Denmark) with an 18-gauge, 25 cm biopsy needle (Bard Max-Core, BD, Franklin Lakes, NJ, USA). In cases with a positive MRI, three to five additional cores were obtained from each suspicious MRI lesion using a US-MRI fusion biopsy system in addition to the standard biopsy. All patients received antibiotic prophylaxis before the procedure. Biopsy specimens were analyzed by two experienced pathologists, blind to patients’ clinical characteristics and following the latest ISUP guidelines [18]. They utilized a four-point scale to classify both Irani G and Irani A. Irani G was categorized as follows: 0—no inflammatory cells present, 1—scattered inflammatory cell infiltrate, 2—non-confluent lymphoid nodules and 3—extensive inflammatory areas with confluent infiltrates. Irani A was defined as 0—no contact between inflammatory cells and glandular epithelium, 1—contact between inflammatory cell infiltrates and glandular epithelium, 2—evident but limited glandular epithelium disruption affecting less than 25% of the examined material and 3—glandular epithelium disruption involving more than 25% of the examined material [9]. The PIS was developed by combining the Irani G and A scores and assessing their frequencies and kernel distribution to determine the most reported clusters. Patients were enrolled after this study was approved by the University of Foggia Ethical Committee (Decision n. 152/CE/2014). Prospectively enrolled patients provided informed consent to be included in our Institutional Database. An institutional review board waiver for informed consent was obtained prior to conducting this study in accordance with institutional regulations when dealing with de-identified previously collected data.

### Statistical Analysis

Continuous variables were reported as median values and interquartile ranges (IQRs) and compared using the ANOVA and Kruskal–Wallis tests. Categorical variables were expressed as percentages and analyzed using the Chi-square test. The association between the inflammation scores (Irani G, Irani A, novel PIS) and the diagnosis of any PCa and clinically significant prostate cancer (csPCa) defined as ISUP Grade Group (GG) ≥ 2 was tested using multivariable logistic regression models, adjusting for age, PSA levels, biopsy history and prostate volume. All statistical tests were performed using Stata-SE 16.1 (StataCorp LP, College Station, TX, USA) and were two-sided, with the significance level set at *p* < 0.05.

## 3. Results

A total of 5091 patients underwent PBx during the study period. Details on number of patients excluded and reason for exclusion are presented in Supplementary Figure S1. The final population consisted of 4620 patients. The detection rate of any PCa and csPCa was 47% and 25%, respectively. The association between the Irani G and A scores and patients’ clinical and pathological characteristics is reported in Table 1.

**Figure 1:**
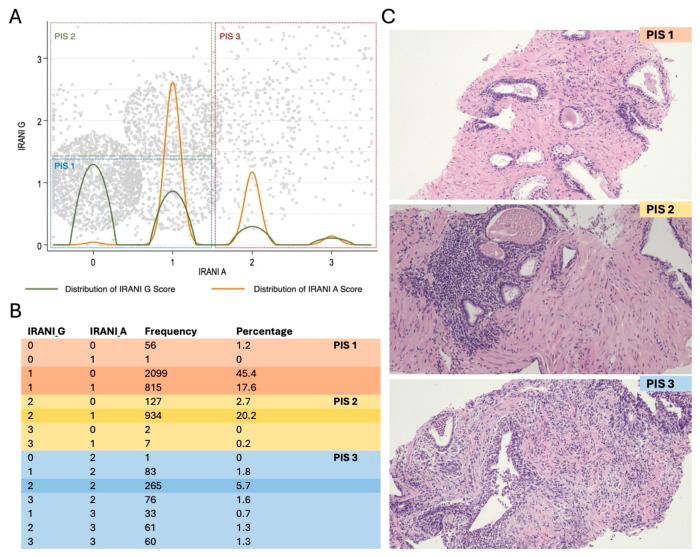
Pattern of presentation of intraprostatic inflammation. (**A**,**B**) Distribution of IRANI G and IRANI A scores and corresponding PIS scores. Each grey dot is a patient. The green line (Irani G) and the orange line (Irani A) represent the kernel density of each subscore. The higher the curve, the higher the number of patients with that score. Most patients had IRANI A 0–1 G 0–1 (PIS 1) or Irani A 0–1 G 2–3 (PIS 2). (**C**) Representative images of intraprostatic inflammation in prostate biopsy cores according to PIS score. (Hematoxylin and Eosin, original magnification 100×). PIS 1: Scattered inflammatory cell infiltrates without lymphoid aggregates; PIS 2: a discrete nodule of mononuclear inflammatory cells focally infiltrating the epithelium; PIS 3: confluent sheets of mononuclear inflammatory cells infiltrating the lumen of glands and ducts.

Patients with low Irani G and Irani A scores were more likely to have PCa and csPCa as compared to patients with high Irani G and A scores. Specifically, the PCa detection rate was 52.5%, 50.8%, 37.3% and 28.7% in patients with low Irani G, low Irani A, High Irani G and high Irani A, respectively. Similar proportions were seen for csPCA (29.3%, 28.8%, 22.3% and 14.7%, respectively). On the other hand, patients with high Irani G scores had lower Qmax and greater PVR and IPSS than those with low Irani G scores, whereas such differences were not seen between patients with high and low Irani A scores. Figure 1 shows the frequencies of the different combinations of Irani G and A scores. Their kernel distribution allowed for the identification of three clusters, specifically, Irani G0/1 with A 0/1, named PIS 1, Irani G2/3 with Irani A 0/1, named PIS 2, and Irani A 2/3 with any Irani G, named PIS 3. Overall, 2971 (64%) patients were classified as PIS 1, 1070 (23%) as PIS 2 and 579 (13%) as PIS 3.

Table 2 summarizes patients’ clinical and pathological characteristics according to the novel PIS. Patients with PIS 1 had significantly (*p* < 0.0001) higher rates of any PCa than those with PIS 2 and PIS 3 (53.8% vs. 40.2% vs. 28.7%), and the same applied for csPCa rates (30% vs. 25.2% vs. 14.7%). On the other hand, patients with PIS 2 had significantly lower Qmax and significantly greater PVR and IPSS.

Multivariable logistic regression analysis (Table 3) confirmed that patients with high Irani G or high Irani A score had a significantly lower risk of being diagnosed with any PCa and csPCa, highlighting difficulties in determining such risks in those with heterogeneous features such as high Irani G and low Irani A scores or vice versa. Conversely, the comprehensive PIS proved that the higher the score, the lower the likelihood of being diagnosed with PCa. Patients with PIS 2 indeed had significantly lower odds of being diagnosed with any PCa (OR = 0.58, 95% CI: 0.48–0.71, *p* < 0.001) and csPCa (OR = 0.75, 95% CI: 0.60–0.94, *p* = 0.010); those with PIS 3 had even lower odds of being diagnosed with any PCa (OR = 0.35, 95% CI: 0.26–0.46, *p* < 0.001) and csPCa (OR = 0.38, 95% CI: 0.27–0.53, *p* < 0.001).

## 4. Discussion

Risk stratification for PCa continues to be a pressing clinical dilemma. This study confirmed that PI may play a role in this stratification. In a large cohort of patients, lower Irani G scores (0–1) and low Irani A scores were associated with an increased risk of PCa diagnosis found on PBx. On the other hand, patients with high Irani G scores had lower Qmax and greater PVR and IPSS than those with low Irani G scores, whereas such differences were not seen between patients with high and low Irani A scores. These findings would suggest that the different types of inflammation patterns leading to high Irani G or high Irani A scores may reflect different clinical conditions. Moreover, as many as 1070 (23.2%) of our 4620 patients had low Irani A with high Irani G scores, and another 106 (2.3%) patients had high Irani A with low Irani G scores, thus making the Irani score suboptimal in scoring PCa risk in patients with heterogeneous features, one every four in the present series. The aim of this study was to evaluate whether a novel comprehensive score obtained by combining Irani G and A scores could better predict the presence of PCa at PBx. Based on Kernel’s distribution, patients with homogeneous Irani features, specifically low (0–1) Irani A and G scores, were classified as PIS 1, and patients with non-homogeneous Irani features, specifically low Irani A and high Irani G scores, were classified as PIS 2, and patients with high Irani A and (mainly high) G scores were classified as PIS 3. We found that the higher the PIS, the lower the likelihood of being diagnosed with PCa or csPCa since PIS 2 and 3 patients had significantly lower odds of being diagnosed with PCa than PIS 1 patients. Looking at pathology findings, the risk of being diagnosed with PCa was highest in patients with limited glandular epithelial and stromal inflammation (PIS 1 = low Irani A and G scores), lower in patients with limited glandular epithelial but significant stromal inflammation (PIS 2 = low Irani A but high Irani G scores) and lowest in those with high glandular epithelial inflammation with or without stromal inflammation (PIS 3 = high Irani A independently on Irani G scores). Therefore, we speculate that the risk of PCa decreases with the degree and type of inflammation. The presence of stromal inflammation in the form of confluent or non-confluent lymphoid nodules (high Irani G) seems to reduce the risk of PCa in patients with low Irani A; on the other hand, the presence of glandular epithelial disruption by the inflammatory infiltrate (high Irani A) seems to be associated with the lowest risk of PCa. Interestingly, there was no difference in PSA levels between PIS 1, 2 and 3 patients, reinforcing the concept that, for the same PSA level, the presence of high G and, even more, high A inflammation reduces the risk of being diagnosed with PCa. The clinical significance of inflammatory cells in the prostate has been considered controversial [19]. One study postulated a direct association between PI and PCa, but it was carried out on patients without clinical indications for PBx who adhered to the prostate cancer prevention trial (PCPT) [20]. Several other studies conversely pointed out an inverse association between PI and PCa. In an autopsy study, Delongchamps et al. reported that chronic inflammatory infiltrates were more prevalent in glands with BPH than in glands without BPH or glands containing cancer [21]. Karakiewicz et al. demonstrated that chronic inflammation present in PBx specimens was associated with an 80% decrease in the probability of receiving a CaP diagnosis [22]. Similarly, Porcaro et al. pointed out that infection in this initial set of random PBxs significantly decreased the relative risk of PCa diagnosis found [23]. In a recent meta-analysis of 25 studies (a total of 20,585 patients tested, of whom 6641 were PCa positive), PI was found to be inversely associated with the risk for malignancy at PBx [24]. Other studies have emphasized the potential protective role of PI for the prevention of PCa. For example, a Finnish PCa detection trial showed that a biopsy specimen diagnosis of inflammation was associated with a reduced risk of subsequent diagnosis of PCa in subsequent biopsies [25]. In addition, Moreira et al., in a REDUCE trial [26] of 6238 patients with a previous negative biopsy, demonstrated a lower risk of CaP at the 2-year follow-up biopsy for men who had had acute or chronic inflammation in baseline biopsy samples. In line with these studies, the present study confirmed the inverse association between PI and PCa. It also showed that PIS 2 patients had the worst BPH-related parameters (prostate volume, Qmax, PVR and IPSS), suggesting BPO, whereas PIS 3 patients had intermediate findings, suggesting chronic inflammatory prostatitis. A strong point of our study was the use of a scoring system for PI, namely the Irani score, which is validated and can be easily carried out during routine pathology examination on hematoxylin and eosin-stained slides. The novelty and further strength of the present study was using the Irani score to develop a comprehensive prostatic inflammation score that could overcome the interpretation problems arising from heterogeneous Irani score findings. Indeed, the novel PIS allowed for the stratification of the risk of being diagnosed with PCa as high (PIS 1), intermediate (PIS 2) and low (PIS 3). This said, our study is not without limitations. First, we did attempt to further correlate PIS and ISUP GG; this issue would deserve further studies since data would suggest that the lower the PIS, the higher the cancer aggressiveness. Second, we did not perform a sub-analysis of patients who had or had not a US/MRI fusion biopsy, but we felt this would have had no impact on the association between PI and the presence of PCa at PBx. Third, it should be acknowledged that this is a retrospective analysis of data prospectively collected at a single center; therefore, external validation is eagerly awaited.

## 5. Conclusions

The development of tools to enhance PCa risk stratification remains a critical clinical challenge. This study highlighted that the PIS obtained during routine pathology examination was significantly associated with the risk of being diagnosed with any PCa and csPCa at PBx. While PI seems to be overall protective over PCa, the different types (stromal vs. glandular) of inflammation depicted by PIS seem to express a different risk.

## Figures and Tables

**Table 1:** Clinical and pathological patients’ characteristics according to Irani G and A score.

	IRANI G 0–1 (N = 3088)	IRANI G 2–3 (N = 1532)	*p* Value	IRANI A 0–1 (N = 4041)	IRANI A 2–3 (N = 579)	*p* Value
Age	67 (61, 72)	67 (62, 72)	0.2	67 (62, 72)	66 (60, 72)	0.008
PSA	6.2 (4.7, 9.1)	6.4 (4.8, 9.7)	0.095	6.3 (4.8, 9.2)	6.4 (4.7, 10.0)	0.7
Biopsy History, n (%)						
Naive	2451 (79.4%)	1167 (76.2%)	0.035	3180 (78.7%)	438 (75.7%)	0.2
Previous Negative	637 (20.6%)	365 (23.8%)		861 (21.3%)	141 (24.3%)	
DRE, n (%)						
Negative	1887 (61.1%)	949 (61.9%)	0.6	2485 (61.5%)	351 (60.7%)	0.8
Suspicious	1201 (38.9%)	583 (38.9%)		1556 (38.5%)	228 (39.3%)	
Prostate volume	50 (36, 66)	56 (40, 78)	<0.0001	50 (37, 70)	53 (40, 75)	0.005
PSA density	0.13 (0.09, 0.21)	0.12 (0.08, 0.19)	0.0004	0.13 (0.08, 0.21)	0.12 (0.08, 0.19)	0.023
Qmax, mL/s	14 (10, 21)	12 (9, 18)	<0.0001	13 (9, 20)	14 (10, 19)	0.9
PVR, mL	20 (1, 50)	30 (1, 50)	<0.0001	30 (1, 50)	20 (1, 50)	0.8
IPSS	9 (4, 15)	11 (6, 18)	<0.0001	10 (5, 16)	10 (5, 17)	0.3
LUTS, n (%)						
Mild (1–7)	1633 (52.9%)	648 (42.3%)	<0.0001	2020 (50.0%)	261 (45.0%)	0.079
Moderate (8–19)	1096 (35.5%)	654 (42.7%)		1497 (37.0%)	253 (43.7%)	
Severe (20–35)	359 (11.6%)	230 (15.0%)		524 (13.0%)	65 (11.3%)	
Biopsy Result, n (%)						
Negative	1465 (47.4%)	961 (62.7%)	<0.0001	2013 (49.8%)	413 (71.3%)	<0.0001
GGG 1	717 (23.2%)	230 (15.0%)		866 (21.4%)	81 (14.0%)	
GGG ≥ 2	906 (29.3%)	341 (22.3%)		1162 (28.8%)	85 (14.7%)	

PSA: prostate-specific antigen; DRE: digitorectal examination; Qmax: peak flow rate; PVR: post-void residual volume; IPSS: International Prostate Symptom Score; LUTS: lower urinary tract symptoms (assessed using the IPSS score); GGG: Gleason Grade group.

**Table 2:** Clinical and pathological patients’ characteristics according to novel PIS.

	Overall N = 4620	PIS 1 (N = 2971)	PIS 2 (N = 1070)	PIS 3 (N = 579)	*p* Value
Age	67 (61, 72)	67 (61, 72)	67 (62, 73)	66 (60, 72)	0.005
PSA	6.3 (4.7, 9.3)	6.2 (4.7, 9.0)	6.4 (4.9, 9.6)	6.4 (4.7, 10.0)	0.2
Biopsy History, n (%)					
Naive	3622 (78.4%)	2356 (79.3%)	824 (77.0%)	438 (75.7%)	0.2
Previous Negative	998 (21.6%)	615 (20.7%)	246 (23.0%)	141 (24.3%)	
DRE, n (%)					
Negative	2837 (61.4%)	1812 (61.0%)	672 (62.8%)	351 (60.7%)	0.7
Suspicious	1783 (38.6%)	1159 (39.0%)	398 (37.2%)	228 (39.3%)	
Prostate volume	50 (37, 70)	50 (36, 65)	57 (40, 80)	53 (40, 75)	<0.0001
PSA density	0.13 (0.08, 0.20)	0.13 (0.09, 0.21)	0.12 (0.08, 0.19)	0.12 (0.08, 0.19)	0.0002
Qmax, mL/s	14 (9, 20)	14 (10, 21)	12 (9, 18)	14 (10, 19)	<0.0001
PVR, mL	30 (1, 50)	20 (1, 50)	30 (1, 50)	20 (1, 50)	<0.0001
IPSS	10 (5, 16)	9 (4, 16)	11 (6, 18)	10 (5, 17)	<0.0001
LUTS, n (%)					
Mild (1–7)	2278 (49.3%)	1575 (53.0%)	446 (41.7%)	261 (45.0%)	<0.0001
Moderate (8–19)	1751 (37.9%)	1043 (35.1%)	454 (42.4%)	253 (43.7%)	
Severe (20–35)	591 (12.8%)	354 (11.9%)	170 (15.9%)	65 (11.3%)	
Biopsy Result, n (%)					
Negative	2426 (52.5%)	1373 (46.2%)	640 (59.8%)	413 (71.3%)	<0.0001
GG 1	947 (20.5%)	706 (23.8%)	160 (15.0%)	81 (14.0%)	
GG ≥ 2	1247 (27.0%)	892 (30.0%)	270 (25.2%)	85 (14.7%)	

PIS: prostatic inflammation score; PSA: prostate-specific antigen; DRE: digitorectal examination; Qmax: peak flow rate; PVR: post-void residual volume; IPSS: International Prostate Symptom Score; LUTS: lower urinary tract symptoms (assessed using the IPSS score); GG: ISUP Grade group.

**Table 3:** Multivariable analysis predicting any PCa and csPCa using the PIS score.

**Multivariable Analysis Predicting Any PCa** **(ISUP GG ≥ 1)**											
	PIS				IRANI G				IRANI A		
Covariate	OR	95% CI	*p* > |z|	Covariate	OR	95% CI	*p* > |z|	Covariate	OR	95% CI	*p* > |z|
Age, per y	1.08	1.07, 1.09	<0.001	Age, per y	1.08	1.07, 1.10	<0.001	Age, per y	1.08	1.07, 1.09	<0.001
PSA, per unit	1.10	1.08, 1.12	<0.001	PSA, per unit	1.10	1.08, 1.12	<0.001	PSA, per unit	1.10	1.08, 1.12	<0.001
Biopsy History, n (%)				Biopsy History, n (%)				Biopsy History, n (%)			
Naive	Ref			Naive	Ref			Naive	Ref		
Previous Negative	0.49	0.40, 0.60	<0.001	Previous Negative	0.49	0.40, 0.60	<0.001	Previous Negative	0.49	0.40, 0.60	<0.001
DRE				DRE				DRE			
Negative	Ref			Negative	Ref			Negative	Ref		
Suspicious	1.66	1.40, 1.97	<0.001	Suspicious	1.66	1.40, 1.97	<0.001	Suspicious	1.64	1.39, 1.95	<0.001
Prostate volume, per mL	0.97	0.97, 0.97	<0.001	Prostate volume, per mL	0.97	0.97, 0.97	<0.001	Prostate volume, per mL	0.97	0.97, 0.97	<0.001
PIS				IRANI G				IRANI A			
1	Ref			0–1	Ref			0–1	Ref		
2	0.58	0.48, 0.71	<0.001	2–3	0.54	0.45, 0.64	<0.001	2–3	0.40	0.30, 0.52	<0.001
3	0.35	0.26, 0.46	<0.001								
**Multivariable Analysis Predicting csPCa** **(ISUP GG ≥ 2)**											
Covariate	OR	95% CI	*p* > |z|	Covariate	OR	95% CI	*p* > |z|	Covariate	OR	95% CI	*p* > |z|
Age, per y	1.08	1.07, 1.09	<0.001	Age, per y	1.08	1.07, 1.09	<0.001	Age, per y	1.08	1.07, 1.09	<0.001
PSA, per unit	1.13	1.11, 1.15	<0.001	PSA, per unit	1.13	1.11, 1.15	<0.001	PSA, per unit	1.13	1.11, 1.15	<0.001
Biopsy History, n (%)				Biopsy History, n (%)				Biopsy History, n (%)			
Naive	Ref			Naive	Ref			Naive	Ref		
Previous Negative	0.41	0.32, 0.53	<0.001	Previous Negative	0.41	0.32, 0.52	<0.001	Previous Negative	0.41	0.32, 0.53	<0.001
DRE				DRE				DRE			
Negative	Ref			Negative	Ref			Negative	Ref		
Suspicious	2.28	1.90, 2.73	<0.001	Suspicious	2.28	1.91, 2.73	<0.001	Suspicious	2.27	1.89, 2.72	<0.001
Prostate volume, per mL	0.97	0.97, 0.97	<0.001	Prostate volume, per mL	0.97	0.97, 0.97	<0.001	Prostate volume, per mL	0.97	0.97, 0.97	<0.001
PIS				IRANI G				IRANI A			
1	Ref			0–1	Ref			0–1	Ref		
2	0.75	0.60, 0.94	0.010	2–3	0.64	0.52, 0.78	<0.001	2–3	0.40	0.29, 0.57	<0.001
3	0.38	0.27, 0.53	<0.001								
**Multivariable Analysis** **(IPSS > 7) (In Patients with a Negative Biopsy)**											
Covariate	OR	95% CI	*p* > |z|	Covariate	OR	95% CI	*p* > |z|	Covariate	OR	95% CI	*p* > |z|
Age, per y	1.00	0.99, 1.02	0.776	Age, per y	1.00	0.99, 1.02	0.813	Age, per y	1.00	0.99, 1.02	0.744
PSA, per unit	0.99	0.97, 1.02	0.683	PSA, per unit	0.99	0.97, 1.02	0.659	PSA, per unit	1.00	0.97, 1.02	0.755
Biopsy History, n (%)				Biopsy History, n (%)				Biopsy History, n (%)			
Naive	Ref			Naive	Ref			Naive	Ref		
Previous Negative	1.03	0.79, 1.33	0.854	Previous Negative	1.02	0.79, 1.33	0.872	Previous Negative	1.03	0.79, 1.33	0.852
Prostate volume, per mL	1.01	1.01, 1.01	<0.001	Prostate volume, per mL	1.01	1.01, 1.01	<0.001	Prostate volume, per mL	1.01	1.01, 1.01	<0.001
PIS				IRANI G				IRANI A			
1	Ref			0–1	Ref			0–1	Ref		
2	1.17	0.90, 1.52	0.234	2–3	1.24	0.98, 1.56	0.077	2–3	1.21	0.87, 1.69	0.255
3	1.28	0.91, 1.80	0.161								

PIS: prostatic inflammation score; PSA: prostate-specific antigen; DRE: digitorectal examination; IPSS: International Prostate Symptom Score; GG: ISUP Grade group.

## Data Availability

The data presented in this study will be made available by the authors on request.

## Supplementary Materials

The following supporting information can be downloaded at: https://www.mdpi.com/article/10.3390/diagnostics15020166/s1, Figure S1: Study flow chart.
