# Supplementary figures and images for: The Novel Histological Prostatic Inflammation Score Helps Defining the Association Between Stromal and Glandular Inflammation with the Risk of Prostate Cancer at Prostate Biopsy
Source: Diagnostics (Basel). 2025 Jan 13;15(2):166. doi: 10.3390/diagnostics15020166 (PMC11763971; doi:10.3390/diagnostics15020166)

## Supplementary Materials

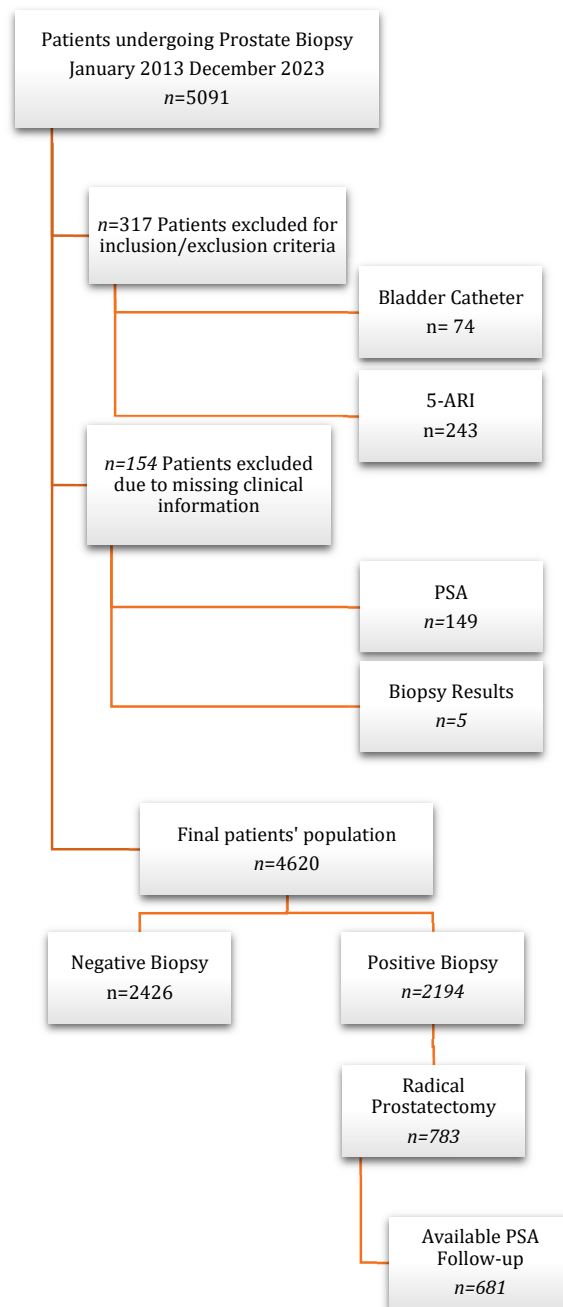

**Figure S1.** Study flow chart.

Supplement: Supplementary file 1 [file diagnostics-15-00166-s001.zip › Supplementary Figure S1.pdf]
